# Association between Peripheral Oxidative Stress and White Matter Damage in Acute Traumatic Brain Injury

**DOI:** 10.1155/2014/340936

**Published:** 2014-04-03

**Authors:** Wei-Ming Lin, Meng-Hsiang Chen, Hung-Chen Wang, Cheng-Hsien Lu, Pei-Chin Chen, Hsiu-Ling Chen, Nai-Wen Tsai, Yu-Jih Su, Shau-Hsuan Li, Chia-Te Kung, Tsui-Min Chiu, Hsu-Huei Weng, Wei-Che Lin

**Affiliations:** ^1^Department of Diagnostic Radiology, Chiayi Chang Gung Memorial Hospital and Chang Gung University College of Medicine, 6 West Section, Chiapu Road, Putzu, Chiayi 61363, Taiwan; ^2^Department of Diagnostic Radiology, Kaohsiung Chang Gung Memorial Hospital and Chang Gung University College of Medicine, 123 Ta-Pei Road, Niao-Sung, Kaohsiung 83305, Taiwan; ^3^Department of Neurosurgery, Kaohsiung Chang Gung Memorial Hospital and Chang Gung University College of Medicine, 123 Ta-Pei Road, Niao-Sung, Kaohsiung 83301, Taiwan; ^4^Department of Neurology, Kaohsiung Chang Gung Memorial Hospital and Chang Gung University College of Medicine, Kaohsiung, Taiwan; ^5^Department of Biomedical Imaging and Radiological Sciences, National Yang-Ming University, Taipei, Taiwan; ^6^Department of Internal Medicine, Kaohsiung Chang Gung Memorial Hospital and Chang Gung University College of Medicine, 123 Ta-Pei Road, Niao-Sung, Kaohsiung 83301, Taiwan; ^7^Department of Emergency Medicine, Kaohsiung Chang Gung Memorial Hospital and Chang Gung University College of Medicine, 123 Ta-Pei Road, Niao-Sung, Kaohsiung 83301, Taiwan

## Abstract

The oxidative stress is believed to be one of the mechanisms involved in the neuronal damage after acute traumatic brain injury (TBI). However, the disease severity correlation between oxidative stress biomarker level and deep brain microstructural changes in acute TBI remains unknown. In present study, twenty-four patients with acute TBI and 24 healthy volunteers underwent DTI. The peripheral blood oxidative biomarkers, like serum thiol and thiobarbituric acid-reactive substances (TBARS) concentrations, were also obtained. The DTI metrics of the deep brain regions, as well as the fractional anisotropy (FA) and apparent diffusion coefficient, were measured and correlated with disease severity, serum thiol, and TBARS levels. We found that patients with TBI displayed lower FAs in deep brain regions with abundant WMs and further correlated with increased serum TBARS level. Our study has shown a level of anatomic detail to the relationship between white matter (WM) damage and increased systemic oxidative stress in TBI which suggests common inflammatory processes that covary in both the peripheral and central reactions after TBI.

## 1. Introduction

Approximately 1.4 million people sustain traumatic brain injury (TBI) in the United States every year [1]. Oxidative stress is believed to be one of the mechanisms involved in the neuronal damage and a close relationship exists between the degree of oxidative stress and the pathogenesis of TBI [2]. Thus, there is compelling clinical need for real-time serum biochemical marker tests to aid in the diagnosis and severity stratification of head injuries.

Lipid peroxidation and inflammatory processes have been shown to increase blood-brain barrier permeability. As an immediate response, vasogenic and cytotoxic edema develop within the first hour after TBI. Thiobarbituric acid reactive species (TBARS) is widely adapted as a sensitive method for measuring lipid peroxidation [3]. In addition, several studies have measured the levels of antioxidants as potential indirect markers of oxidative stress in brain injury. Plasma concentrations of ascorbic acid, *α*-tocopherol, and protein thiols are likewise associated with the degree of neurological impairment [4]. Biochemical marker testing that provides prognostic information on short-term patient outcome, especially among mild TBI cases, will be immensely valuable for patient management.

Diffuse axonal injury (DAI) and cortical contusions constitute the vast majority of primary intra-axial lesions in cases of TBI and are associated with significant morbidity. During TBI, the subcortical white matter, internal capsule, corpus callosum, fornix, and infratentorial white matter (brain stem and cerebellum) are the most common predicted regions of brain injury [5]. Conventional computed tomography (CT) and standard magnetic resonance imaging (MRI) often underestimate the extent of white matter damage after TBI [6]. Advances in MRI techniques has allowed for the visualization of changes, particularly after a patient's earlier exposure to TBI [7]. The MRI diffusion tensor imaging (DTI) is potentially more sensitive for detecting intracranial microstructural change. Among various quantitative metrics such as apparent diffusion coefficient (ADC) and fractional anisotropy (FA), eigenvalues derived from DTI have been recognized as most useful for evaluating the integrity of white matter fibers [8].

To date, the relations of a panel of inflammatory markers and MRI DTI findings in acute TBI patients have not been examined. Under the hypothesis that increased systemic inflammatory biomarkers are associated with loss of anatomic integrity, this study measured DTI metrics at the deep brain regions in TBI patients to evaluate their correlation with serum inflammatory biochemical marker levels.

## 2. Material and Methods

### 2.1. Patients

Sixty-three patients who sustained TBI between June and December 2012 and admitted at Kaohsiung Chang Gung Memorial Hospital were enrolled. The diagnosis of acute TBI was confirmed by history and brain CT scans. All of the patients underwent brain CT scan shortly after arriving at the emergency room. Repeat brain CT scan or/and MRI were performed for any clinical deterioration (e.g., acute-onset focal neurologic deficits, seizures, status epilepticus, and progressively disturbed consciousness) and as routine postneurosurgical procedure. After complete neurologic examination and history taking, the patients were under continuous observation and monitored regularly for Glasgow Coma Scale (GCS) Score, electrocardiogram, blood pressure, pulse rate, temperature, fluid balance, and laboratory parameters.

On initial CT study, patients with massive epidural/subdural hemorrhage that could distort the brain tissue or with any parenchymal lesion that might affect diffusion tensor MRI results were excluded. Those with the following were also excluded: (1) age < 20 years; (2) under medication with antiplatelet or anticoagulant drugs before the acute TBI; (3) having evidence of alcoholism, any other addictive disorders, or known affective or other psychiatric diseases than those caused by sedatives or neuroleptics; (4) having known neurologic disorders potentially affecting the central nervous system; and (5) having major systemic diseases like end-stage renal disease, liver cirrhosis, or congestive heart failure.

Among the 63 patients, 25 had at least one of massive epidural hematoma (EDH), subdural hematoma (SDH), or subarachnoid hematoma (SAH) that caused anatomical structure distortions, while 11 had minor EDH, SDH, or SAH but combined with parenchymal contusion hematoma. Two patients were excluded due to alcoholism and one for age < 20 years. Twenty-four patients with TBI were finally included in this study. For diffusion tensor MRI and biomarker comparison, 24 age- and sex-matched healthy volunteers were also enrolled. The Ethics Committee of the hospital's Institutional Review Board approved the study and all participants provided written informed consent.

### 2.2. Laboratory Measurements for Oxidative Stress Factors

#### 2.2.1. Blood Sampling

All subjects received blood sampling in day one after TBI (for the patients) or when normal controls were enrolled in the study. Sera were isolated from peripheral blood samples drawn from each subject before and after the expedition. Blood samples were centrifuged at 3000 rpm for 10 minutes. Each serum sample was collected and frozen at −80°C prior to biochemical measurements.

#### 2.2.2. Determination of Serum Thiobarbituric Acid-Reactive Substances

Thiobarbituric acid-reactive substances (TBARS) was measured based on a well-established method for detecting lipid peroxidation [3]. The TBARS Assay Kit is a standard tool to assaying the the lipid perioxidation level in serum. Under high temperature (90–100°C) and acidic condition measure thiobarbituric acid-malondialdehyde (TBA-MDA) level via colorimeteorically at 532 nm as described by the manufacturer (cat. 10009055; Cayman 4 Chemical). Briefly, serum (100 mL) was added in duplicate to sodium dodecyl sulfate (SDS) (100 mL) and color reagent (4 mL). These mixtures were then incubated for 1 hour in boiling water and centrifuged at 1600 g for 10 min at 4°C. After warming for 5 min at 25°C, the samples were read on a microplate spectrophotometer (Beckman Coulter). Values for samples were calculated from a linear calibration curve prepared using pure MDA-containing samples (range, 0–50 *μ*mol/L).

#### 2.2.3. Determination of Serum Free Thiol Content

The ability of antioxidative defense in response to increased oxidative damage was evaluated by measuring the serum level of total reduced thiols since serum thiols were physiologic free radical scavengers. Serum total protein thiols were estimated by directly reacting thiols with 5,5-dithiobis 2-nitrobenzoic acid (DTNB) to form 5-thio-2-nitrobenzoic acid (TNB). The amount of thiols in the sample was calculated from the absorbance determined using the extinction coefficient of TNB (A412 = 13,600 M^−1^ cm^−1^).

### 2.3. MRI Acquisition

MRI was performed in all subjects in the same day of blood sampling. Subjects were examined for DTI study in day one after TBI (for the patients) or when normal controls were enrolled in this study. The DTI datasets were acquired by using single-shot diffusion spin-echo, echo-planar imaging with a TR/TE of 15 800 millisecond/minimum, a 2.5 mm section, a matrix of 128 × 128, number of excitations of 3, and an FOV of 25.6 × 25.6 cm, yielding an in-plane resolution of 2 mm, with a total acquisition time of 12 min. The DTI encoding entailed 13 noncollinear directions, with *b* = 1000 s/mm^2^, and a nondiffusion T2-weighted image. Contiguous sections (*n* = 55) were obtained without an intersection gap to achieve total cerebral coverage.

The DTI sets were transferred to an offline workstation for further analysis using the FuncTool diffusion tensor protocol (Advanced Workstation 4.2; GE Healthcare), which contained a preprocessing function to remove echo-planar imaging distortions like scaling, shearing, and translation due to eddy current effects from a diffusion gradient. The distortion-corrected data were then interpolated to attain isotropic voxels and decoded to obtain the tensor field for each voxel. The algorithm computed the 6 coefficients of the diffusion tensor for each pixel location. The tensor field data was then used to compute the DTI metrics, including the mean diffusivity (ADC) and FA for each voxel.

### 2.4. DTI Data Analysis

**Figure 1:**
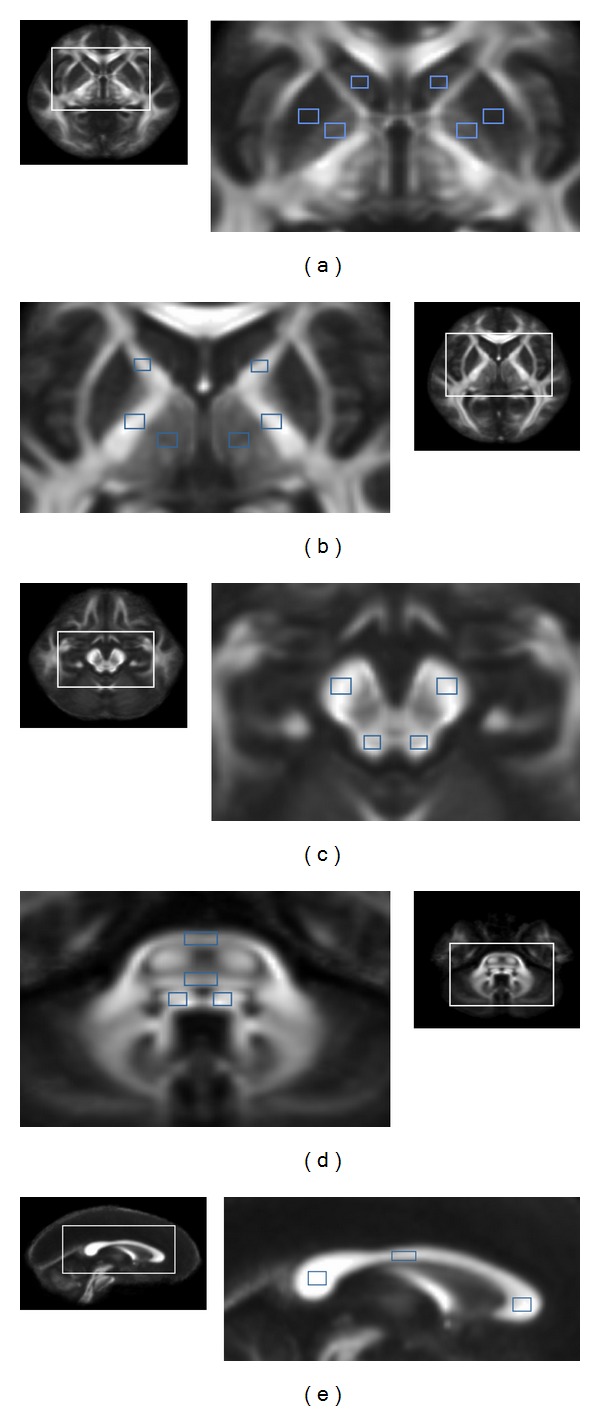
Regions of interest were placed on the (a) caudate, putamen, and globus pallidum; (b) the anterior and the posterior limbs of the internal capsule, thalamus; (c) cerebral peduncles, superior cerebellar peduncles; (d) middle cerebellar peduncles, pontine crossing tract, and medial lemniscus; (e) the genu, body, and splenium of corpus callosum.

Regions of interest, 50–100 mm^2^ depending on the anatomic region, were measured by a radiologist and confirmed by another radiologist to avoid malpositioning (Figure 1). The measurements were performed according to the method described by Lin et al. [9, 10]. Regions of interest were placed on the caudate, putamen, globus pallidum, anterior and posterior limbs of the internal capsule, thalamus, cerebral peduncles, superior and middle cerebellar peduncles, pontine crossing tract, medial lemniscus, and the genu, body, and splenium of the corpus callosum.

The first level of the region of interest was selected at the foramen of Monro. At this level, regions of interest at the caudate, putamen, globus pallidum, and thalamus were measured on two continuous sections (Figures 1(a) and 1(b)). At the same level, the anterior internal capsule (bounded by the head of the caudate nucleus and the globus pallidus) and the posterior internal capsules (defined by the globus pallidus and thalamus) were measured on two contiguous sections (Figure 1(b)).

The second level was selected at the inferior colliculus and the regions of interest at the cerebral peduncle and superior cerebellar peduncle and measured on two continuous sections (Figure 1(c)). The third level was selected at the trigeminal nerve origin from the pons and the regions of interest at the middle cerebellar peduncle, pontine crossing tract, and medial lemniscus were measured on two continuous sections (Figure 1(d)). Regions of interest at the genu, body, and splenium of the corpus callosum were placed on three consecutive sections of median sagittal section on which their full volume was obtained (Figure 1(e)). Cerebral structures were carefully identified on T1- and T2-weighted images to avoid partial volume averaging due to CSF. Regions of interest were drawn on a null image of DTI and were automatically transferred onto FA and ADC maps for each subject. The average FA and ADC of 22 regions of interest were calculated. The raters were blinded to the subjects' details.

### 2.5. Statistical Analysis

The Statistics Package for Social Science, Version 17.0 (SPSS Inc, Chicago, IL, USA) software, was used to perform all statistical analyses. The Student's *t*-test and the chi-square test were applied to compare the age and sex between groups, respectively. Multivariate analysis of covariance (MANCOVA) model with age and sex as covariates was used to investigate differences in serum oxidative stress factors such as serum thiol and TBARS concentrations between the two groups. Post-hoc tests with Bonferroni's correction were performed for multiple comparisons. Statistical significance was set at *P* < 0.05.

The MANCOVA model with age and sex as covariates was also used to investigate differences in the diffusivity indices of the regions of interest between the two groups. Post-hoc tests with Bonferroni's correction were performed for multiple comparisons. The DTI indices between the two groups were significant when *P* was <0.05.

To access the correlation between serum oxidative stress factors and DTI-related indices for the regions of interest, Pearson's partial correlation analysis with age and sex as confounding covariates was performed. To further investigate the relationships among TBI severity, serum oxidative stress factors, and DTI-related indices for regions of interest, the patients were graded based on their GCS, with a GCS > 12 as grade 1, GCS 9–12 as grade 2, and GCS < 9 as grade 3. Pearson's partial correlation analysis with age and sex as confounding covariates was again performed. Statistical significance was set at *P* < 0.05.

## 3. Results

### 3.1. Patients

The demographic and clinical information were listed in Table 1. There were no significant differences between two groups in age (*P* = 0.164, Student's *t*-test) and sex (*P* = 1, chi-square test).

The initial clinical symptoms of the patients with TBI were motor deficit (*n* = 3), posttraumatic amnesia (*n* = 5), and brief unconsciousness. There were no seizure attacks. The imaging findings showed minimal focal epidural hematoma (*n* = 5), subdural hematoma (*n* = 12), subarachnoid hemorrhage (*n* = 12), and pneumocraniun (*n* = 4). There was no depressed skull fracture among these patients. Two received ventriculostomy and craniotomy. With initial GCS of 13.74 ± 3.018, the hospitalization duration reached 11.70 ± 7.945 days, with 3.87 ± 4.506 days in the ICU. One of the patients suffered new-onset neurologic deficit, while two had deterioration of consciousness on followup.

There were significant differences in oxidative stress factors, including serum thiol and TBARS concentrations, between the two groups. The serum TBARS concentration of patients with TBI was significantly higher than those of the controls (*P* = 0.014). There was only borderline difference between two groups in serum thiol concentration (*P* = 0.050).

### 3.2. FA and ADC Values of DTI

**Figure 2:**
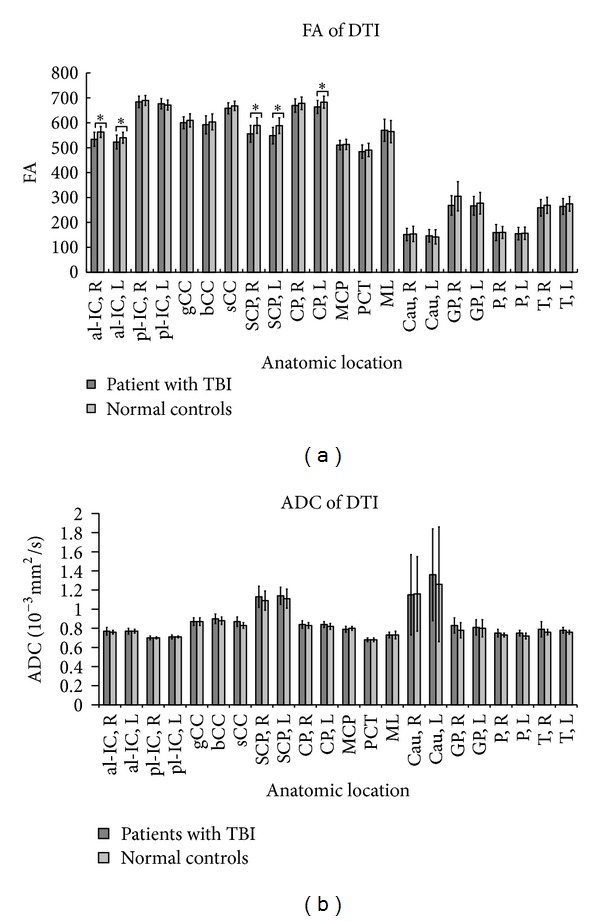
The (a) FAs and (b) ADC of patients compared to the healthy controls. The al-IC and pl-IC represent the anterior and posterior limbs of the internal capsule, respectively, while the gCC, bCC, and sCC represent the genu, body, and splenium of the corpus callosum. The attached R/L represents the right/left side. *Significant difference between the patients and controls (*P* < 0.05). SCP: superior cerebellar peduncle; CP: cerebral peduncle; MCP: mean middle cerebellar peduncle; PCT: pontine crossing tract; ML: medial lemniscus.

The FAs were significantly reduced in patients with TBI compared to those of the controls in the anterior limbs of the bilateral internal capsule, bilateral superior cerebellar peduncles, and left cerebral peduncle (Figure 2). In patients with TBI, the FA was also reduced in most of the regions with abundant WM, including the posterior limb of the internal capsule, whole corpus callosum, right cerebral peduncle, middle cerebellar peduncle, pontine crossing tract, and medial lemniscus, although the differences were not significant (see Supplementary Material available online at http://dx.doi.org/10.1155/2014/340936). There was no significant FA difference between the patients with TBI and normal controls in gray matter (Figure 2(a)). There was no significant ADC difference between the patients with TBI and normal controls in all ROI measured (Figure 2(b)).

### 3.3. Oxidative Stress Factors in relation to FA and ADC Values of DTI and Disease Severity

**Figure 3:**
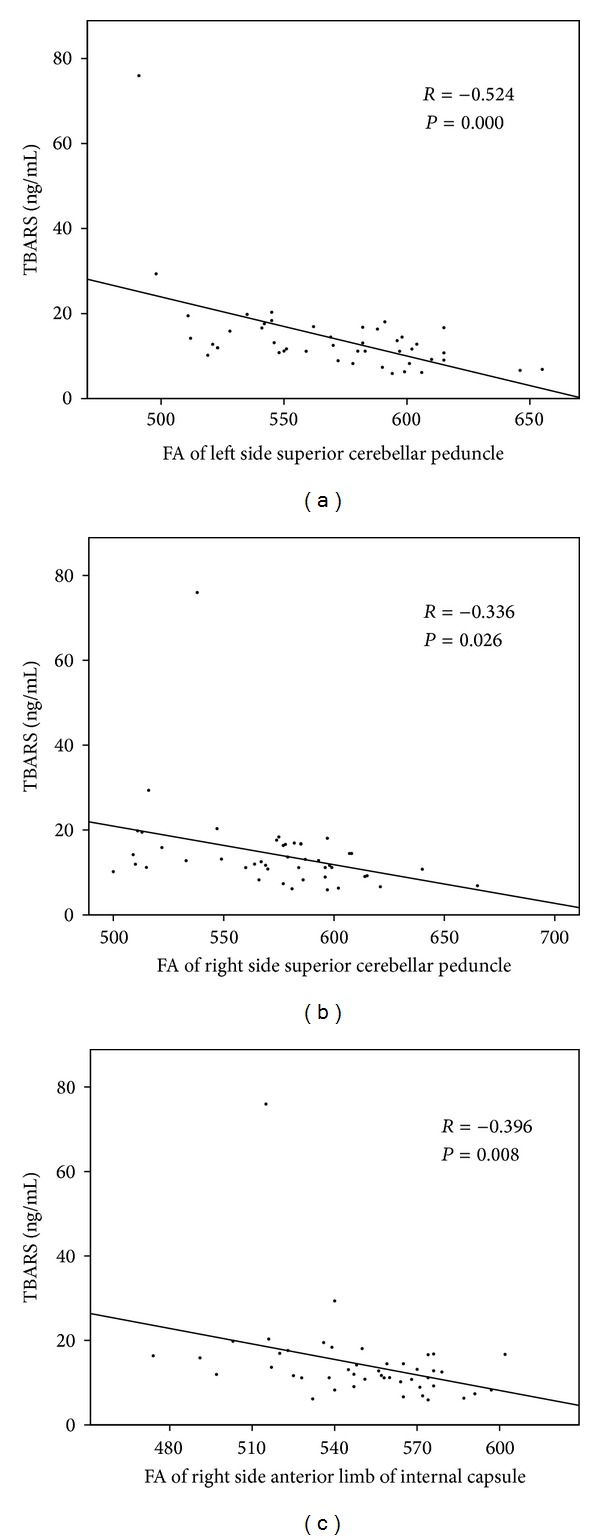
Increased serum TBARS concentration correlated with the decreased FAs in the (a) left (*r* = −0.524, *P* = 0.000) and (b) right (*r* = −0.336, *P* = 0.026) superior cerebellar peduncles and (c) the right anterior limb of the internal capsule (*r* = −0.396, *P* = 0.008).

There was significant correlation between serum TBARS concentration and the FAs of DTI (Figure 3). Decreased FA in the left (*r* = −0.524, *P* = 0.000) and right (*r* = −0.336, *P* = 0.026) superior cerebellar peduncles and the right anterior limb of the internal capsule (*r* = −0.396, *P* = 0.008) were associated with higher serum TBARS concentrations (Figures 3(a)–3(c)). The serum oxidative stress factors or FAs of DTI did not correlate with the initial GCS of the patients. The serum TBARS concentration was not significantly correlated with ADC in any region.

## 4. Discussion

This is the first study to show correlations between serum inflammatory markers and changes of DTI metrics in patients with TBI using DTI metrics. There is a higher systemic TBAR level in patients with TBI than in healthy controls. The study also confirms the hypothesis that anatomic integrity subsequently decreases in the acute phase of the disease, even without visible injury on conventional imaging studies, and that serum TBAR values can predict the degree of DTI metrics degradation in TBI.

After the initial injury in TBI, neuronal damage or death produce proinflammatory substances and generate free radicals [11]. Enhanced oxidative stress that increased production of free radicals has been implicated in the disruption of neuronal homeostasis induced by TBI [12]. One of the major products of lipid peroxidation is TBARS, which is measured in a well-established method for detecting oxidative stress biomarkers [13]. As in previous reports [14], this study demonstrates significantly higher TBARS plasma levels in patients with TBI group than in healthy controls, supporting the pathophysiology of oxidative stress during TBI.

Similar to other antioxidant biomarkers like ascorbic acid, *α*-tocopherol, a protein thiol, has been associated with the degree of neurologic impairment [4]. Thiols are organic sulfur derivatives characterized by the presence of sulfhydryl residues at the active site. Halliwell and Gutteridge have demonstrated that protein-associated thiols, particularly in the albumin molecule, constitute a major defense against oxidative stress in plasma [15]. A previous study has shown that protein-thiol level is a valuable tool for monitoring treatment effects on oxidative stress after TBI [16]. In the present study, however, there is only borderline group difference in serum thiol level. Since patients with minor injury in the acute stage of the disease are the subjects of this investigation, the effect of thiol consumption may be less or insignificant.

Another important finding is the changes of microstructure integrity in the deep brain WM after TBI. By quantification of tissue water diffusion [17], diffusion tensor imaging (DTI) had been demonstrated to be a good imaging modality to detect invisible architectural lesions after TBI [18]. Acceleration and deceleration in TBI can result in traumatic axonal injury and is the possible cause of impaired clinical outcomes [19]. Changes in DTI metrics, such as decreased FA, reflect damage to the WM integrity [18] either by increased radial diffusivity or by decreased axial diffusivity in a diffusion tensor ellipsoid model. The results here regarding TBI with deep brain WM injury, including the internal capsule and brain stem, are consistent with those of previous studies [5]. During TBI, the subcortical white matter, internal capsule, corpus callosum, fornix, and infratentorial white matter (brain stem and cerebellum) are the most common predicted regions with brain injury.

In present study, we did not found significant DTI metrics change in gray matter. Differences in GM and WM diffusion after TBI could be due to variability between these two tissue types at any stage in the process that leads from injury to altered diffusion. Gray matter has been traditionally considered to be more vulnerable than white matter to ischemia [20]. However, more recent findings in experimental models have demonstrated that DTI measurements from the WM occur earlier and with greater severity then previous appreciated even with short periods of ischemia [21]. In addition, decrease diffusion, including reduced bulk water motion from cytoskeletal breakdown and disruption of fast axonal transport [22, 23] that does not present in gray matter, might occur in WM. Our results support previous findings that WM is more vulnerable than gray matter after TBI (diffuse axonal injury) which allows water diffusion to occur more easily in the directions perpendicular to the fiber, thus lowering FA.

The most important finding in the present study is the bridge between central white matter injury and systemic inflammation after TBI. Although the true pathophysiologic mechanism is unclear, several explanations may be posited. Animal model studies and substantial clinical data suggest that blood-brain barrier breakdown frequently follows head trauma [24]. Thom et al. have provided information on nearly 350 proteins associated with central nervous system (CNS) myelin and on the alteration of myelin basic protein due to lipid peroxygenation leading to autoimmunologic demyelination of the CNS [25]. Autoimmunologic demyelination induces further inflammation in the WM. A previous study suggests that myelin basic protein is released into the CSF and later into the general circulation after acute neurologic events [26]. Yamazaki et al. also found a correlation between serum MBP levels and the severity of TBI in patients with acute head injury [27]. Breakdown of the blood-brain barrier may also partially contribute to the elevated serum inflammatory biomarkers after WM injury.

In contrast, leukocyte infiltration from serum into the CNS after breakdown of the blood-brain barrier can further damage neurons by releasing cytotoxic substances [28]. Glial activation, leukocyte recruitment, and upregulation and secretion of mediators like cytokines and chemokines are the characteristic of posttraumatic cerebral inflammatory change [29]. Excessive oxidative stress generation has also been implicated as mediators of demyelination and axonal damage [30].

Unfortunately, neither increased TBARS nor decreased WM integrity correlate with disease severity in the present study. The study cohort is predominantly lesser affected patients, and thus the proteins may be less or have insignificant response after injury. The relatively small number of subjects in a cross-sectional study may also alter the interpretation. Although DTI is well suited for evaluating WM integrity, whether demyelination, axonal injury, or both lead to lower FA values in the present study remains unclear. Further studies with more diffusivity parameters analyses, such as radial diffusivity or axial diffusivity in diffusion tensor ellipsoid model, are needed. A longitudinal study of the present cohort may yield different relations between TBI and inflammatory markers that are more consistent with previous findings.

In conclusion, increased TBARS level and decreased WM integrity in vulnerable brain areas are found in patients with TBI. Possible interactions between peripheral inflammation and CNS microstructural damage likely represent the acute pathologic processes in TBI.

## Supplementary Material

In patients with TBI, the DTI FAs change found in the abundant WM regions and more significant in the bilateral anterior internal capsules, bilateral cerebellar peduncles, and left cerebral peduncle compared to healthy volunteers. However, in the DTI MDs, there are no significant finding compared to healthy volunteers.

## Figures and Tables

**Table 1:** Demographic data of patients with traumatic brain injury (TBI) and healthy controls.

	Patients with TBI	Normal controls	*F*	*P*
Numbers	24	24		
Sex (male/female)	11/13	12/12		1.000
Age (age)	42.79 ± 15.56	42.67 ± 12.68	2.003	0.164
Serum thiol concentration	1.63 ± 0.27	1.45 ± 0.35	4.065	0.050
Serum TBARS concentration	18.24 ± 13.70	10.66 ± 3.244	6.558	0.014*
Initial Glasgow Coma Scale	13.74 ± 3.02			
Motor deficit	3 (12.5%)			
Posttraumatic amnesia	5 (20.8%)			
Seizure	0 (0%)			
Brief unconsciousness	7 (29.2%)			
Depressed skull fracture	0 (0%)			
Pneumocranium	4 (16.7%)			
Traumatic subarachnoid hemorrhage	12 (50%)			
Subdural hematoma	12 (50%)			
Epidural hematoma	5 (20.8%)			
Parenchymal contusion hematoma	0 (0%)			
Operation	2 (8.3%)			
Ventriculostomy	2 (8.3%)			
Craniectomy	0 (0%)			
Craniotomy	2 (8.3%)			
Days of total hospitalization	11.70 ± 7.95			
Days of intensive care unit	3.87 ± 4.51			
Newly onset of neurological deficit	1 (4.2%)			
Deterioration of consciousness	2 (8.3%)			

Data are demonstrated as means ± standard deviation.

*Significant differences (*P* < 0.05).
