# Supplementary material for: Association between Peripheral Oxidative Stress and White Matter Damage in Acute Traumatic Brain Injury
Source: Biomed Res Int. 2014 Apr 3;2014:340936. doi: 10.1155/2014/340936 (PMC3996315; doi:10.1155/2014/340936)
Supplement: Supplementary file 1 — In patients with TBI, the DTI FAs change found in the abundant WM regions and more significant in the bilateral anterior internal capsules, bilateral cerebellar peduncles, and left cerebral peduncle compared to healthy volunteers. However, in the DTI MDs, there are no significant finding compared to healthy volunteers. [file 340936.f1.doc]

Supplementary Table

| DTI FA | Patients with TBI | | | Normal controls | | | F | P |
| --- | --- | --- | --- | --- | --- | --- | --- | --- |
| **Anterior limb of internal capsule R** | **0.53379** | ± | **0.02842** | **0.56302** | ± | **0.02205** | **20.448** | **0.000** |
| **Anterior limb of internal capsule L** | **0.52277** | ± | **0.02785** | **0.53992** | ± | **0.02181** | **7.050** | **0.011** |
| Posterior limb of internal capsule R | 0.68407 | ± | 0.02298 | 0.68953 | ± | 0.02013 | 0.843 | 0.364 |
| Posterior limb of internal capsule L | 0.67626 | ± | 0.02083 | 0.67119 | ± | 0.02010 | 0.906 | 0.346 |
| Genu of corpus callosum | 0.60023 | ± | 0.02375 | 0.60953 | ± | 0.02637 | 1.979 | 0.167 |
| Body of corpus callosum | 0.59215 | ± | 0.03629 | 0.60348 | ± | 0.03199 | 1.432 | 0.238 |
| Splenium of corpus callosum | 0.65834 | ± | 0.02203 | 0.66815 | ± | 0.01857 | 3.148 | 0.083 |
| **Superior cerebellar peduncle R** | **0.55572** | ± | **0.03369** | **0.58922** | ± | **0.03123** | **12.915** | **0.001** |
| **Superior cerebellar peduncle L** | **0.54839** | ± | **0.03318** | **0.58831** | ± | **0.03146** | **18.097** | **0.000** |
| Cerebral peduncle R | 0.66939 | ± | 0.02664 | 0.67804 | ± | 0.02545 | 1.359 | 0.250 |
| **Cerebral peduncle L** | **0.66372** | ± | **0.02574** | **0.68295** | ± | **0.02424** | **7.32** | **0.008** |
| Middle cerebellar peduncle | 0.51046 | ± | 0.01864 | 0.51324 | ± | 0.02060 | 0.244 | 0.623 |
| Pontine crossing tract (a part of MCP) | 0.48430 | ± | 0.02686 | 0.49073 | ± | 0.02665 | 0.691 | 0.410 |
| Medial lemniscus | 0.56982 | ± | 0.04409 | 0.56450 | ± | 0.04439 | 0.165 | 0.686 |
| Caudate R | 0.15140 | ± | 0.02468 | 0.15389 | ± | 0.03066 | 0.097 | 0.757 |
| Caudate L | 0.14626 | ± | 0.02508 | 0.14183 | ± | 0.02887 | 0.318 | 0.575 |
| Globus Pallidum R | 0.26826 | ± | 0.03910 | 0.30501 | ± | 0.05896 | 6.473 | 0.015 |
| Globus Pallidum L | 0.26666 | ± | 0.03762 | 0.27728 | ± | 0.04346 | 0.873 | 0.355 |
| Putamen R | 0.15956 | ± | 0.03216 | 0.15981 | ± | 0.02390 | 0.002 | 0.969 |
| Putamen L | 0.15503 | ± | 0.02525 | 0.15645 | ± | 0.02471 | 0.046 | 0.831 |
| Thalamus R | 0.25875 | ± | 0.03290 | 0.26869 | ± | 0.03271 | 1.074 | 0.306 |
| Thalamus L | 0.26403 | ± | 0.03175 | 0.27450 | ± | 0.02934 | 1.352 | 0.251 |

| DTI MD | Patients with TBI | | | Normal controls | | | F | P |
| --- | --- | --- | --- | --- | --- | --- | --- | --- |
| Anterior limb of internal capsule R | 0.00077 | ± | 0.00004 | 0.00076 | ± | 0.00002 | 0.350 | 0.557 |
| Anterior limb of internal capsule L | 0.00077 | ± | 0.00003 | 0.00077 | ± | 0.00002 | 0.050 | 0.824 |
| Posterior limb of internal capsule R | 0.00070 | ± | 0.00002 | 0.00070 | ± | 0.00001 | 0.278 | 0.601 |
| Posterior limb of internal capsule L | 0.00071 | ± | 0.00002 | 0.00071 | ± | 0.00001 | 0.000 | 1.000 |
| Genu of corpus callosum | 0.00087 | ± | 0.00004 | 0.00087 | ± | 0.00004 | 0.020 | 0.888 |
| Body of corpus callosum | 0.00090 | ± | 0.00005 | 0.00088 | ± | 0.00004 | 3.082 | 0.086 |
| Splenium of corpus callosum | 0.00087 | ± | 0.00005 | 0.00083 | ± | 0.00003 | 6.961 | 0.011 |
| Superior cerebellar peduncle R | 0.00113 | ± | 0.00011 | 0.00109 | ± | 0.00010 | 1.968 | 0.168 |
| Superior cerebellar peduncle L | 0.00114 | ± | 0.00009 | 0.00111 | ± | 0.00010 | 1.092 | 0.302 |
| Cerebral peduncle R | 0.00084 | ± | 0.00004 | 0.00083 | ± | 0.00003 | 1.244 | 0.271 |
| Cerebral peduncle L | 0.00084 | ± | 0.00003 | 0.00082 | ± | 0.00003 | 2.518 | 0.120 |
| Middle cerebellar peduncle | 0.00079 | ± | 0.00003 | 0.00080 | ± | 0.00002 | 0.148 | 0.702 |
| Pontine crossing tract (a part of MCP) | 0.00068 | ± | 0.00002 | 0.00068 | ± | 0.00002 | 1.011 | 0.320 |
| Medial lemniscus | 0.00073 | ± | 0.00003 | 0.00073 | ± | 0.00004 | 0.054 | 0.818 |
| Caudate R | 0.00115 | ± | 0.00042 | 0.00116 | ± | 0.00039 | 0.010 | 0.921 |
| Caudate L | 0.00136 | ± | 0.00048 | 0.00126 | ± | 0.00060 | 0.487 | 0.489 |
| Globus Pallidum R | 0.00083 | ± | 0.00008 | 0.00078 | ± | 0.00008 | 5.003 | 0.030 |
| Globus Pallidum L | 0.00081 | ± | 0.00008 | 0.00080 | ± | 0.00009 | 0.512 | 0.478 |
| Putamen R | 0.00075 | ± | 0.00004 | 0.00073 | ± | 0.00002 | 4.885 | 0.032 |
| Putamen L | 0.00075 | ± | 0.00003 | 0.00072 | ± | 0.00003 | 8.399 | 0.006 |
| Thalamus R | 0.00079 | ± | 0.00008 | 0.00076 | ± | 0.00003 | 2.609 | 0.113 |
| Thalamus L | 0.00078 | ± | 0.00003 | 0.00076 | ± | 0.00002 | 5.672 | 0.022 |
